# Electrical and Anatomical Demonstrations

**Published:** 1887-09

**Authors:** 


*Electrical and Anatomical Demonstrations.* Delivered at the School of Massage and Electricity, Welbeck Street, London. By HERBERT TIBBITS, M.D. A Handbook for trained Nurses and Masseuses. London: J. & A. CHURCHILL. 1887.

The development of the profession of Massage and Electricity demands new text-books for the use of those intending to follow these new avenues to employment. Dr. Tibbits has printed the lectures which he delivered at his hospital to those studying the application of electricity to medicine, and has thereby provided a book which will prove of great use. The various forms of battery and varieties of electricity are clearly explained, and illustrated with good diagrams to render the text more intelligible to those who have not had the advantage of attending his demonstrations. In a separate chapter there is also given a sketch of human anatomy so far as it is requisite for the intelligent use of electricity in medicine. Dr. Tibbits' audiences were composed partly of medical men, as well as of lay-students; so that the book is well worthy of perusal by all practitioners. Hitherto there have been obtained no great therapeutical results from electricity by members of the profession generally; this largely depends upon their imperfect understanding of electrical apparatus and its application in the treatment of disease, a defect which this book can do much to remedy.

